# The relationship of pre-pregnancy body mass index with maternal anthropometric indices, weight retention and the baby’s weight and nutrition in the first 6 months post-partum

**DOI:** 10.1186/s12884-023-06116-0

**Published:** 2023-11-20

**Authors:** Ayda Ahmadibeni, Parhoon Kashani, Mohammad Sadegh Hallaj, Saeed Ghanbari, Nahid Javadifar

**Affiliations:** 1 Student Research Committee, Ahvaz Jundishapur University of Medical Sciences, Ahvaz, Iran; 2 Biostatistics Department, Ahvaz Jundishapur University of Medical Sciences, Ahvaz, Iran; 3 Reproductive Health Promotion Research Center, Ahvaz Jundishapur University of Medical Sciences, Ahvaz, Iran

**Keywords:** Pre-Pregnancy body mass index, Anthropometric Indices, Maternal, Infant

## Abstract

**Purpose:**

Pre-pregnancy body fat mass is one of the important indicators of the mother's and the infant's health. Therefore, the purpose of this study was to investigate relationship of pre-pregnancy body mass index (PPBMI) with maternal anthropometric indices and weight retention as well as the baby's weight and nutrition in the first 6 months post-partum.

**Materials and methods:**

This is a prospective cohort study including 397 mothers giving birth to healthy babies and referring to health centers in Ahvaz (southwest of Iran) in 2022. The following data were extracted from the participants' electronic record: body mass index (BMI) before or at the beginning of pregnancy, gestational weight gain, and weight at the time of delivery. In addition to demographic information, the following data were also evaluated: maternal anthropometric indices including weight, hip and waist circumference, and conicity index during the first 10 days post-partum, along with the weight and nutrition pattern of the baby 2, 4 and 6 months post-partum.

**Results:**

The mean age of the mothers was 29.96±5.7 years. The frequency of mothers according to BMI classification (i.e., underweight, normal, overweight, and obese) was 4.3%, 38.5%, 37%, and 20.3%, respectively. In this study, PPBMI had a significant relationship with decreasive changes of weight, waist and hip circumferen and conicity index after child birth, 2, 4 and 6 months post-partum (*P*<0.05) but the mean reduction of these anthropoemetric indices at 6^th^ month postpartum were not related to PPBMI (*P*>0.05). However, this relationship was not significant when it came to the weight of the baby (*P* > .05). The lowest reduction in weight, waist and hip circumference and conicity index belonged to overweight mothers but the highest frequency of mothers with excesive gestational weight gain, the lowest frequency of breastfeeding until 6 months and also the lowest values of postpartum weight retention were observed in obese mothers (*P*<0.05).

**Conclusion:**

According to the findings of this study, the decrease in anthropometric indices up to 6 months after delivery in overweight mothers is less than other BMI groups, but the consequences related to weight and nutrition in infants of obese mothers need special attention. Also, the results re-emphasize the importance of focusing on provision of educational and counseling services to mothers in order to improve their nutrition and weight, especially before pregnancy.

## Introduction

Pregnancy is one of the most important stages of women's life and a period characterized with increased probability of obesity [1]. Gestational weight gain (GWG) is an important indicator for maternal and infant health and quality of life [2]. However, recent reports indicate high rates of GWG in women with no return to pre-pregnancy weight [3]. The most basic index for obesity is the body mass index (BMI), which is accepted by both the World Health Organization (WHO) and the International Institute of Medicine (IOM) as a measure of obesity [4]. Since 1990, the IOM has provided recommendations regarding GWG based on pre-pregnancy body mass index (PPBMI) in order to optimize fetal growth and postpartum outcomes, and these recommendations were revised in 2009 [4, 5]. Based on PPBMI, women are divided into four categories, namely: Underweight (less than 18.4), Normal (18.5 to 24.9), Overweight (25 to 29.9) and Obese (≥30). According to IOM, the optimal GWG for the underweight mothers is 12.5 to 18 kg, and it is 11.5- 16 kg for those in normal category, 7 to 11.5 kg for overweight mothers, and 5 to 9 kg for obese mothers [4]. A previous study showed that 25% of obese women gained weight according to IOM recommendations, 27% gained insufficient weight, and 48% exceeded the recommended values [6].

Optimal GWG is essential for optimal maternal and fetal outcomes because inappropriate GWG can pose health risks to both the mother and the baby [7]. Evidence has shown that women who are overweight or obese before pregnancy are more at risk of adverse maternal or neonatal outcomes, especially the risk of low birth weight (LBW) or macrosomia [8], which are the main causes of child mortality [9]. In a normal process at birth, the weight of the baby, the amniotic fluid and the placenta are lost, and by week 6, the blood volume is reduced to pre-pregnancy levels, and the uterus returns to its normal size. The excess weight remaining after this period comes mainly from body fat reserves. Accordingly, mothers experience a significant weight loss during the first 6 weeks after birth, especially in the first 2 to 3 weeks post-partum [10]. Research shows that failure to lose or maintain the GWG after child birth will lead to maternal overweight and obesity [11]. The results of a study have shown that 75% of women are heavier one year after giving birth than what they weighed before pregnancy, with nearly 50% of them maintaining more than 10 pounds and almost 25% maintaining more than 20 pounds [12]. Also, the findings of a study in Vietnam have shown that being underweight before pregnancy and experiencing excessive weight gain (EWG) help to retain weight up to one year after delivery [13].

Although BMI has been widely used as an anthropometric index, it fails to explain the distribution of fat mass, and studies have shown that complications due to obesity are more associated with abdominal fat. For this reason, in recent years, measurements of waist circumference, the ratio of waist circumference to hip circumference, and height have been considered as alternatives to BMI in clinical research. Recently, the conicity index has also been suggested to check the obesity status, and a number of studies have reported its superior value compared to the ratios of waist circumference to hip and waist circumference to height [14, 15].

Despite the large body of literature on the relationship between PPBMI and advers pregnancy or neonatal outcomes [8, 9], there is dearth of studies on the relationship between PPBMI and changes in anthropometric indices, conicity index, and weight retention in mothers as well as the baby's weight and nutrition up to 6 months after delivery [3, 16]. Therefore, the focus of the present study was to evaluate these relationships.

## Materials and methods

This is a prospective cohort research approved by the Ethics Committee of Ahvaz Jundishapur University of Medical Sciences (IR.AJUMS.REC.1399.188). In Ahvaz city, there are a total of 36 comprehensive health service centers (19 centers are located in the east and 17 centers are located in the west of the city). In this study, a total of 8 centers were randomly selected (4 centers from the west and 4 centers from the east districts of Ahvaz). After receiving approval from university, the research team visited the selected health centers and obtained the approval of the relevant authorities. Then the eligible participants were gradually selected from among mothers referring to those centers in a simple random sample. The number of women selected from each center was proportional to the population under coverage. Based on the findings of a previous study [4], the sample size was calculated to be 388 using MedCalc ® statistical software with 80% power and 5% error. In 2022, a total of 600 pregnant mothers were examined of whom only 397 remained, and the rest were gradually excluded from the study based on the exclusion criteria.

$$\begin{array}{l}n=\frac{{\left({z}_{1-\frac{\alpha }{2}}+{z}_{\beta }\right)}^{2}}{{\left(0.5 ln\frac{1+r}{1-r}\right)}^{2}}+3\\ \begin{array}{l}\alpha =0.05\gg \gg {z}_{1-\alpha /2}=1.96\\ \beta =0.02\gg \gg {z}_{\beta }=1.28\\ r=0.142\end{array}\end{array}$$

The inclusion criteria in the present study were: willingness to participate in the study, the presence of information about weight before or at the beginning of pregnancy, GWG, birth weight and breastfeeding, age over 18 years, and having a healthy singleton baby. Exclusion criteria were: unwillingness to continue participation in the study, maternal or infantile chronic diseases and medical conditions requiring medical interventions, the presence of high-risk pregnancy (preeclampsia, gestational diabetes, depression, placenta previa, and twin pregnancy), addiction to drugs such as psychotropic or stimulant drugs, consuming alcoholic beverages, smoking cigarettes or hookah, or being on maternal post-partum weight loss diet or professional exercise programs.

After the participants were briefed on the study objectives, informed consent was obtained from them, and they were requested to complete a demographic and obstetrics questionnaire. The content validity of this questionnaire was approved by 15 faculty members of Ahvaz Jundishapur University of Medical Sciences, Ahvaz. Information about weight and BMI before or at the beginning of pregnancy (up to 13 weeks of pregnancy) as well as GWG was extracted from the women's electronic record and was divided and recorded based on IOM classification.

Upon the first postnatal visit (within the first 10 days after delivery), the weight, waist circumference, hip circumference, and conicity of the mother were measured using a standard scale in the center (which was previously calibrated with a standard weight) and a plastic tape measure. The above indicators and the weight of the child were measured and recorded again 2, 4 and 6 months after delivery as part of the routine care of the child. The mother's weight was measured with minimal clothing and barefooted with the same scale (which was previously calibrated with a standard weight). Abdominal circumference was measured after several consecutive normal exhalations and was based on the middle point of the distance between the pelvic spine and the last palpable rib and around the buttock at the level parallel to the floor and at the place of the largest part of the pelvis, using an inflexible plastic tape measure without imposing any pressure on the person's body which was covered by light clothing. This was done following the protocol of the World Health Organization. Then, using the formula: $conicity=\frac{wc (m)}{0.109\sqrt{\frac{ht (m)}{wt (kg)}}}$, the anthropometric index of conicity, which indicates the amount of fat and obesity status, was calculated and recorded [17]. Also, by subtracting the initial weight of the participant before pregnancy or at the beginning of pregnancy until the 13th week from the weight of 6 months after delivery, we obtained the weight retention.

Statistical analysis was done using SPSS version 20. Descriptive statistics (mean, standard deviation, frequency, percentage, etc.), Chi-square test, ANOVA and repeated measure test were used to compare characteristics between different groups. Statistical significance was set at *p*<0.05.

## Results

The participants of the present study included 397 pregnant mothers whose mean age was 30 years. The body mass index before or at the beginning of pregnancy (PPBMI) indicated underweight in 17 (4.3%) mothers, normal weight in 153 (38.5%), overweight in 147 (37%), and obesity in 80 (20.2%) women. Table 1 shows the relationship between body mass index and four demographic variables, namely mother's age, educational attainment, occupation, and family income. Among the above variables, the mother's age and educational attainment had a significant relationship with body mass index (*P*<0.05). The majority of mothers (57.4%) were over 30 years and mother's age has a significant relationship with different body mass indices, with the body mass index increasing with age. In this study, the majority of the participants (46.1%) had a university degree, and the highest percentage of these mothers (44.3%) were in the body mass index group above normal. As far as family income was concerned, 84.9% of the participants reported that their family income was sufficient.

**Table 1:** The relationship between maternal PPBMI and mother's demographic status

Qualitative Variables		BMI					Pearson chi-square	*p*-value
		**Less than normal**	**Normal**	**Above normal**	**Fat**	**Total**		
**Mother age (yr)**	18–24	9 (12.7%)	36 (50.7%)	20 (28.2%)	6 (8.5%)	71 (17.9%)	33.89	<0.001
	25–29	5 (5.1%)	44 (44.9%)	30 (30.6%)	19 (19.4%)	98 (24.7%)		
	>30	3 (1.3%)	73 (32%)	97 (42.5%)	55 (24.1%)	228 (57.4%)		
**Mother Education**	High school	8 (8.2%)	36 (37.1%)	29 (29.9%)	24 (24.7%)	97 (24.4%)	14.91	.021
	Diploma	6 (5.1%)	47 (40.2%)	37 (31.6%)	27 (23.1%)	117 (29.5%)		
	College education	3 (1.6%)	70 (38.3%)	81 (44.3%)	29 (15.8%)	183 (46.1%)		
**Mother Job**	Governmental employee	0 (0.0%)	13 (40.6%)	14 (43.8%)	5 (15.6%)	32(8.1%)	11.75	.068
	Housewife	17 (4.9%)	136 (39.1%)	121(34.8%)	74(21.3%)	348(87.7%)		
	Non-governmental employee	0 (0.0%)	4 (23.5%)	12 (70.6%)	1(5.9%)	17(4.3%)		
**Income**	Enough	14 (4.2%)	134 (39.8%)	120 (35.6%)	69 (20.5%)	337 (84.9%)	2.28	.516
	Insufficient	3 (5.0%)	19 (31.7%)	27 (45.0%)	11 (18.3%)	60 (15.1%)		

Chi-square test was used for all analyses

Table 2 provides information about the following quantitative variables: anthropometric indicators of weight, waist circumference, hip circumference and conicity index of the mother and baby's weight in 4 evaluations at birth, 2, 4 and 6 months post-partum. Table 3 includes the values of weight retention rate (weight of 6 months after delivery—initial weight before or at the beginning of pregnancy), maternal weight reduction (weight after childbirth – weight at 6th month), waist circumference reduction ( waist circumference after chilbirth—waist circumference at 6th month), hip circumference reduction (hip circumference after chilbirth—hip circumference at 6th month), conicity index reduction (conicity index after chilbirth—conicity index at 6th month) and baby weight gain ( baby weight at 6th month—baby birth weight) in mothers with body mass indices indicating underweight, normal weight, overweight, and obese.

**Table 2:** The relationship of maternal PPBMI with age, anthropometric indices, and baby weight

*Mother Weight (Kg)*					
Pre pregnancy BMI groups (n)	After child birth Mean ± SD	2 months after child birth Mean ± SD	4 months after child birth Mean ± SD	6 months after child birth Mean ± SD	*Within subject effect*
Less than normal (17)	56.65 ± 7.71	55.71 ± 9.38	54.08 ± 8.93	52.75 ± 8.72	F = 110.61 *P* < 0.001
Normal (153)	65.09 ± 8.85	63.09 ± 8.01	62.04 ± 7.68	60.95 ± 7.47	
Above normal (147)	76.00 ± 8.48	74.05 ± 8.03	73.01 ± 7.74	72.36 ± 7.29	
Fat (80)	87.99 ± 8.05	85.68 ± 7.98	84.76 ± 8.14	83.66 ± 8.69	
*Between subject effect*	F = 179.5 *P* < 0.001				
*Mother Waist Circumference (Cm)*					
Less than normal (17)	86.12 ± 13.81	81.47 ± 13.27	79.65 ± 11.93	75.88 ± 10.60	F = 242.84 *P* < 0.001
Normal (153)	92.77 ± 11.86	88.25 ± 10.44	86.33 ± 10.04	84.73 ± 10.07	
Above normal (147)	103.09 ± 9.63	98.53 ± 8.71	96.73 ± 8.88	95.10 ± 8.56	
Fat (80)	109.86 ± 10.46	104.01 ± 9.30	101.79 ± 9.65	100.03 ± 10.10	
*Between subject effect*	F = 69.35 *P* < 0.001				
*Mother Hip Circumference (Cm)*					
Less than normal (17)	94.53 ± 11.63	92.68 ± 12.12	91.35 ± 12.10	89.24 ± 10.69	F = 104.79 *P* < 0.001
Normal (153)	103.33 ± 10.80	99.77 ± 9.96	98.34 ± 9.73	96.97 ± 9.29	
Above normal (147)	112.44 ± 7.90	108.49 ± 7.50	106.95 ± 7.05	105.55 ± 7.14	
Fat (80)	119.48 ± 7.43	114.72 ± 7.18	112 ± 7.34	111.49 ± 7.16	
*Between subject effect*	F = 75.81 *P* < 0.001				
*Mother Conicity Index*					
Less than normal (17)	1.35 ± 0.19	1.28 ± 0.15	1.27 ± 0.14	1.23 ± 0.13	F = 169.58 *P* < 0.001
Normal (153)	1.35 ± 0.13	1.30 ± 0.11	1.28 ± 0.11	1.27 ± 0.12	
Above normal (147)	1.38 ± 0.11	1.34 ± 0.09	1.32 ± 0.10	1.31 ± 0.10	
Fat (80)	1.37 ± 0.12	1.31 ± 0.10	1.29 ± 0.11	1.28 ± 0.12	
*Between subject effect*	F = 4.21 *P* = 0.006				
*Baby Weight (Gr)*					
Less than normal (17)	3146.76 ± 370.67	5026.47 ± 516.64	6417.65 ± 788.38	7544.12 ± 924.30	F = 3046.75 *P* < 0.001
Normal (153)	3184.38 ± 469.50	5186.44 ± 756.36	6614.87 ± 911.47	7627.58 ± 967.56	
Above normal (147)	3233.06 ± 441.15	5220.95 ± 687.32	6705.34 ± 843.67	7808.30 ± 951.50	
Fat (80)	3340.00 ± 456.24	5258.13 ± 682.31	6591.760 ± 65	7641.88 ± 907.94	
*Between subject effect*	F = 0.890 *P* = 0.446				

Repeated measure test was used for all analyses

**Table 3:** Mean and Standard deviation of postpartum weight retention, reduction of anthropometric indices and baby weight gain in pre pregnancy BMI groups

Pre pregnancy BMI groups	Postpartum weight retention (Kg) Mean ± SD	Mother weight redu*ctio*n (Kg) Mean ± SD	Mother Waist Circumference reduction (Cm) Mean ± SD	Mother Hip Circumference reduction (Cm) Mean ± SD	Mother Conicity Index reduction Mean ± SD	Baby weight gain (Gr) Mean ± SD
Less than normal	6.45 ± 6.42	3.9 ± 4.15	10.23 ± 7.50	5.30 ± 5.60	0.11 ± 0.10	4247.65 ± 622.62
Normal	1.89 ± 4.12	4.14 ± 4.31	8.04 ± 6.48	6.35 ± 5.11	0.07 ± 0.07	4193.68 ± 759.64
Above normal	0.82 ± 4.27	3.64 ± 4.33	7.99 ± 6.43	6.28 ± 9.86	0.07 ± 0.71	4181.08 ± 954.60
Fat	-0.81 ± 5.22	4.34 ± 5.08	9.84 ± 5.78	7.99 ± 5.38	0.09 ± 0.73	4113 ± 730.37
F (P-value)	14.39 (< 0.001)	0.52 (0.668)	2.188 (0.089)	1.296 (0.276)	2.16 (0.092)	0.222 (0.0881)

ANOVA test was used for all analyses

According to the results of repeated measure test in Table 2, PPBMI had a significant relationship with decreasive changes of weight, waist and hip circumferen and conicity index after child birth, 2, 4 and 6 months post-partum (*P*<0.05). As can be seen in Table 3, the greatest amount of weight loss in six months after delivery was in obese wemen, followed by women with normal weight, underweight, and overweight mothers. The order of hip circumference decrease from highest to lowest was in obese, normal weight, overweight, and underweight mothers, respectively. The same order for waist circumference and conicity index was: underweight, obese, normal weight, and overweight mothers, respectively. If we disregard the small number of mothers with an PPBMI representing underweight (*n*=17) compared to other groups, the greatest reductions in waist circumference, hip circumference, and conicity index six months after delivery were observed in obese and normal weight mothers, respectively but overweight mothers experienced the lowest decrease. These relationships were not significant. Table 2 shows that there is no significant relationship between PPBMI of the mothers and the weight of the baby at birth, and 2, 4, and 6 months after birth. However, the highest to lowest baby weight gain up to six months after birth was seen in underweight, normal, overweight and obese mothers, respectively (Table 3). According to this table, maternal weight retention sixth months after delivery was significantly different in mothers with different body mass index classifications, with the highest rate of weight retention being seen in underweight mothers (6.45 kg). Interestingly, not only did obese mothers experience no weight retention, but some of them experienced weight loss (0.8 kg) compared with their weight recorded in the first pregnancy visit.

Table 4 shows the relationship between BMI and the following qualitative variables: GWG, different values of maternal weight maintenance in the sixth month after delivery, parity, weight at birth, and the predominant feeding pattern of the baby up to six months of age.

**Table 4:** The relationship of PPBMI with maternal total weight gain, postpartum weight retention, parity, baby's birth weight, and baby nutrition pattern

Qualitative Variables		BMI					Person chi-square	p-value
		Less than normal	Normal	Above normal	Fat	Total		
Total Weight gain	Inadequate	5(4.8%)	52(49.5%)	27(25.7%)	21(20.0%)	105(26.4%)	31.16	< 0.001
	Adequate	7(5.1%)	64(47.1%)	49(36.0%)	16(11.8%)	136(34.3%)		
	Excessive	5(3.2%)	37(23.7%)	71(45.5%)	43(27.6%)	156(39.3%)		
Retained Postpartum Weight	< 1.5 kg (n %)	4(1.8%)	83(36.9%)	87(38.7%)	51(22.7%)	225(56.7%)	47.55	< 0.001
	1.5-3kg (n %)	5(6.7%)	27(36.0%)	29(38.7%)	14(18.7%)	75(18.9%)		
	3–4.5 kg (n %)	0(0.0%)	8(30.8%)	8(30.8%)	10(38.5%)	26(6.5%)		
	4.5–6 kg (n %)	0(0.0%)	15(51.7%)	14(48.3%)	0(0.0%)	29(7.3%)		
	> 6kg (n %)	8(19.0%)	20(47.6%)	9(21.4%)	5(11.9%)	42(10.6%)		
Para	1–2	16 (5.6%)	123(42.9%)	114(39.7%)	34(11.8%)	287(72.3%)	46.84	< 0.001
	3–4	1(1.0%)	27(26.7%)	31(30.7%)	42(41.6%)	101(25.4%)		
	> 4	0(0.0%)	3(33.3%)	2(22.2%)	4(44.4%)	9(2.3%)		
Birth Weight	< 2500	2(8.3%)	10(41.7%)	9(37.5%)	3(12.5%)	24(6.0%)	3.37	.761
	2500- 4000	15(4.2%)	139(38.5%)	134(37.1%)	73(20.2%)	361(90.9%)		
	> 4000	0(0.0%)	4(33.3%)	4(33.3%)	4(33.3%)	12(3.0%)		
Baby Food	Breast milk	17(5.4%)	122(38.6%)	116(36.7%)	61(19.3%)	316(79.6%)	16.43	.012
	Breast milk & Formula	0(0.0%)	31(39.7%)	31(39.7%)	16(20.5%)	78(19.6%)		
	Formula	0(0.0%)	0(0.0%)	0(0.0%)	3(100%)	3(0.8%)		

Chi-square test was used for all analyses

In this study, gestational weight gain had a significant relationship with PPBMI, so that with increasing BMI, EWG or excesive weight gainl also increased.

PPBMI was significantly associated with maternal weight retention rate. In underweight mothers, the majority retained a weight of more than 6 kg. In normal weight, overweight, and obese mothers, on the other hand, the majority retained a weight of less than 1.5 kg. Also, BMI had a significant relationship with parity, with higher parities indicating higher BMIs. The highest number of mothers para 3 and above was seen in obese mothers. In this study, the birth weight of the baby had no significant relationship with maternal BMI, and the number of macrosomic babies (birth weight 4 kg and above) was equal in all three groups of normal weight, overweight, and obese. According to Table 4, baby's nutrition pattern had a significant relationship with maternal BMI. The predominant nutrition pattern of most babies up to their 6 months of age in all four different groups of maternal BMI was exclusive breastfeeding. The predominant nutrition pattern in normal and overweight mothers was a combination of breastfeeding and baby formula in 7.8% of cases, while this rate was only 4% in the obese mothers. A very small minority of babies born to obese mothers (0.8%) were fed exclusively with baby formula.

## Discussion

The purpose of this study was to investigate the relationship of PPBMI with changes in maternal anthropometric indices, conicity index, postpartum weight retention and also baby's weight and nutrition up to 6 months after delivery. In this study, PPBMI had a significant relationship with decreasive changes of weight, waist and hip circumferen and conicity index after child birth, 2, 4 and 6 months post-partum (*P*<0.05) but the mean reduction of these anthropoemetric indices at 6^th^ month postpartum were not related to PPBMI (*P*>0.05). However, this relationship was not significant when it came to the weight of the baby (*P* > 0.05). The lowest reduction in weight, waist and hip circumference and conicity index belonged to overweight mothers but the highest frequency of mothers with excesive gestational weight gain, the lowest frequency of breastfeeding until 6 months and also the lowest values of postpartum weight retention were observed in obese mothers(*P*<0.05).

While BMI is a good indicator to show the nutritional status of the mother, it simply shows crude obesity and can neither distinguish fat-free mass or muscle mass from body fat mass nor show the distribution of fat in the body [17, 18]. Moreover, body fat percentage can vary significantly between different people with the same BMI [19]. Hence, conicity index, which includes weight, height and waist circumference variables, has been introduced as a suitable index for evaluating central or abdominal obesity. High conicity index is associated with diseases such as diabetes, hypertension, and atherosclerosis [20]. However, it is said that about 30% of women with normal BMI at the beginning of pregnancy will have abdominal obesity after giving birth and are exposed to its complications [21].

In this study, the greatest decrease in anthropometric indices such as weight and hip circumference, was observed in obese mothers, while the lowest decrease was in overweight women. Also, overweight mothers gained more weight than the recommended values during pregnancy compared with other mothers. Therefore, it can be argued that overweight mothers in the present study gained more weight during pregnancy than did other mothers, and they were less likely to experience reduced anthropometric indices six months after delivery. Although there is no global consensus on the optimal amount of GWG based on initial BMI, especially in obese mothers [22], a number of studies have shown that the higher the PPBMI, the lower the GWG [23, 24]. Of course, a previous study found that obese mothers gained more weight during pregnancy [25], which is not consistent with the results of the present study.

It has been reported that mothers with GWG above the recommended values may retain up to 7 kg of post-partum weight [26]. In this study, the majority of underweight mothers (35.3%) retained more than 6 kg of post-partum weight whereas the majority of normal (54%), overweight (59.1%) and obese women (63.75%) retained less than 1.5 kg of post-partum weight. Therefore, it could be argued that the mean retained weight six months after delivery has an inverse relationship with the mother's PPBMI, with obese mothers having the lowest weight retention rate after delivery. In line with the results of the present study, the findings of Jayasinghe et al. (2022) showed that obese women had the lowest rate of weight retention after delivery [27]. However, Sobhan et al. (2019) found that mothers whose GWG was more than the recommended amount, regardless of the mother's initial BMI, had more weight retention, which is not in agreement with the findings of the present study [3]. The results of another study have also indicated that the postpartum weight retention is an important factor in the occurrence of obesity up to one year after childbirth, even for women who had a normal weight before childbirth [28].

Along with other studies, the results of this study showed that the increase in the initial BMI classification of the mother was directly related to the frequency of LBW babies (11.7%, 6.5%, 6.1% and 3.7%, respectively) and had an inverse relationship with macrosomia (0%, 2.6%, 2.7% and 5%, respectively) although these relationships were not significant [27, 28].

In this study, although the birth weight of the baby at 2, 4, and 6 months of age was not significantly different between the groups of mothers' PPBMI, in line with many previous studies, the baby's birth weight rose with an increase in the mother's initial BMI [29–33]. The same pattern was also maintained 2 months after delivery, but at 4 and 6 months post-partum, the highest weight was seen in babies born to overweight mothers [16]. Although very limited studies have addressed the relationship between the mother's initial BMI and the baby's weight in infancy, it is reported that the nutritional status of the mother during pregnancy causes lasting changes in the structural and physiological metabolic functions of the baby [34]. Gul et al. (2020) believe that the reason for the strong relationship between the mother's initial BMI and the weight of the baby is the existence of an intrauterine fetal programming mechanism that causes a higher birth weight which becomes more prominent as the child gets older regardless of the effect of lifestyle and nutrition of the mother and the family [30].

Despite WHO's recommendation for exclusive breast feeding (EBF) until the child is 6 months old [35], the results of a meta-analysis have shown that with the increase in the mother's BMI, mothers are more likely to avoid EBF, stop it early, or discontinue it before the baby is 6 months old [36]. Such a trend can be observed in the results of the present study. Ballesta-Castillejos et al. (2020) point out several reasons for the lower probability of EBF in mothers with higher BMI, namely the higher probability of pregnancy complications, caesarean section, less skin-to-skin contact, the delay in lactogenesis due to the stability of progesterone level in fat tissues, and lack of proper milk flow owing to the anatomical characteristics of the breast tissue in obese mothers [37].

In this study, of all investigated demographic indicators, the mother's age, educational attainment, and parity had a significant relationship with her PPBMI. It is well established that the risk of obesity increases with age [38], which can be caused by fat storage over many years [39]. Also, the number of pregnancies increases with the age of the mother. Therefore, the results of this study are in line with previous studies that have shown that higher parity is directly related to a higher BMI at the beginning of pregnancy [38, 39]. Also, the relationship between mother's PPBMI and her education has been confirmed in previous studies [40]. It has been argued that education represents the culture and social background of the family while income shows the current social status of the person [41]. Although in this study no correlation was found between income level and initial BMI, the results of a study in France showed that obesity and the mother's overweight are inversely related to education and family income level [42].

## Strengths and weaknesses

There are a number of factors which make the present study particularly worthwhile, namely the prospective design of the study, the use of the conicity index and other anthropometric indicators of the mother, and the assessment of the weight and nutrition of the baby at different intervals up to 6 months after delivery. Few, if any, studies have thus far addressed these factors. However, the most important limitation of this study is that in prenatal care, only weight and body mass index are measured, and there is no information about other anthropometric indicators such as waist circumference, hip circumference, and conicity before or at the beginning of pregnancy in mothers cases. Another limitation is the classification of mothers' BMI based on the cut-off points provided by WHO for Western countries, which limits the generalizability of its results to mothers of Asian countries and other races [43]. Also, eating habits and culture can affect the anthropometric indices of the mother and baby and thus affect the comparison of results.

## Conclusion

In this study, the greatest decrease in postpartum weight reduction and hip circumference were observed in women whose PPBMI indicated obesity. These mothers also had the greatest frequency of excesive gestational weight gain, the lowest baby weight gain and exclusive breastfeeding up to 6 months after childbirth. The greatest reduction in postpartum weight retention, waist circumference and conicity index were in underweight mothers while the lowest decrease was observed in overweight women.

Also, the results of the present study re-emphasized the importance of focusing on provision of educational and counseling services to mothers to improve their nutrition and weight, especially before trying to get pregnant. In addition, considering that in many countries, the basis for classifying body mass index and weight gain during pregnancy is the WHO’s recommendations, it seems that it is necessary to conduct more research to modify and adapt the cut-off points based on race and ethnicity.

## Data Availability

The databases used and analyzed are available from the corresponding author on reasonable request.

## Abbreviations

PPBMI: Pre-pregnancy body mass index
BMI: Body mass index
LBW: Low birth weight
GWG: Gestational weight gain
WHO: World Health Organization
IOM: International Institute of Medicine
EWG: Excessive weight gain
Kg: Kilogram

## References

[1] Ring LE. Obesity in pregnancy. Consult Obstet Anesthesiol. 2018;419–22.

[2] Gou BH, Guan HM, Bi YX, Ding BJ (2019). Gestational diabetes: Weight gain during pregnancy and its relationship to pregnancy outcomes. Chin Med J (Engl).

[3] Subhan FB, Shulman L, Yuan Y, McCargar LJ, Kong L, Bell RC, et al. Association of pre-pregnancy BMI and gestational weight gain with fat mass distribution and accretion during pregnancy and early postpartum: a prospective study of Albertan women. BMJ Open. 2019;9(7):0–9. 10.1136/bmjopen-2018-026908 PMC6661681 31352413

[4] Nartea R, Mitoiu BI, Nica AS (2019). Correlation between Pregnancy Related Weight Gain, Postpartum Weight loss and Obesity: a Prospective Study. J Med Life.

[5] Toro-Ramos T, Heaner M, Yang Q, Deluca L, Behr H, Reynolds K (2021). Postpartum weight retention: a retrospective data analysis measuring weight loss and program engagement with a mobile health program. J Women’s Heal.

[6] Waring ME, Moore Simas TA, Liao X (2013). Gestational weight gain within recommended ranges in consecutive pregnancies: a retrospective cohort study. Midwifery.

[7] Asefa F, Cummins A, Dessie Y, Hayen A, Foureur M (2020). Gestational weight gain and its effect on birth outcomes in sub-Saharan Africa: systematic review and meta-analysis. PLoS One.

[8] Tela FG, Bezabih AM, Adhanu AK (2019). Effect of pregnancy weight gain on infant birth weight among mothers attending antenatal care from private clinics in Mekelle City, Northern Ethiopia: a facility based follow-up study. PLoS One.

[9] Wang X, Zhang X, Zhou M, Juan J, Waang X (2019). Association of prepregnancy body mass index, rate of gestational weight gain with pregnancy outcomes in Chinese urban women. Nutr Metab.

[10] McKinley MC, Allen-Walker V, McGirr C, Rooney C, Woodside JV (2018). Weight loss after pregnancy: challenges and opportunities. Nutr Res Rev.

[11] Huseinovic E, Bertz F, Brekke HK, Winkvist A (2018). Two-year follow-up of a postpartum weight loss intervention: results from a randomized controlled trial. Matern Child Nutr.

[12] Minkovitz CS, Schetter CD. Obesity at one year. Obstet Gynecol. 2016;125(1):144–52. 10.1097/AOG.0000000000000565 PMC4286308 25560116

[13] Van Ha AV, Zhao Y, Pham NM, Nguyen CL, Nguyen PTH, Chu TK (2019). Postpartum weight retention in relation to gestational weight gain and pre-pregnancy body mass index: a prospective cohort study in Vietnam. Obes Res Clin Pract.

[14] Sardinha LB, Santos  DA, Silva AM, Gr A (2016). . A Comparison between BMI , Waist Circumference , and Waist-To-Height Ratio for Identifying Cardio-Metabolic Risk in Children and Adolescents.

[15] Gowda V, Philip KM. Abdominal volume index and conicity index in predicting metabolic abnormalities in young women of different socioeconomic class. Int J Med Sci Public Health. 2016;5(4):1452–6.

[16] Łoniewska B, Michalczyk K, Podsiadło K, Adamek K, Michalczyk B, Tousty P, et al. Analysis of the influence of pre-pregnancy BMI and weight gain during pregnancy on the weight of healthy children during the first 2 years of life: a prospective study. Children. 2022;9(10):0–14. 10.3390/children9101431 PMC9600872 36291367

[17] Valdez R (1991). A simple model-based index of abdominal adiposity. J Clin Epidemiol.

[18] Nuttall FQ (2015). Body mass index: obesity, BMI, and health: a critical review. Nutr Today.

[19] Chen G, Arthur R, Iyengar NM, Kamensky V, Xue X, Wassertheil-smoller S, et al. Association between regional body fat and cardiovascular disease risk among postmenopausal women with normal body mass index. Eur Heart J. 2019;40(34):2849–55. 10.1093/eurheartj/ehz391 PMC6933870 31256194

[20] Andrade MD, de Freitas MCP, Sakumoto AM, Pappiani C, de Andrade SC, Vieira VL (2016). Association of the conicity index with diabetes and hypertension in Brazilian women. Arch Endocrinol Metab.

[21] Cherono R, Ogada IA, Kimiywe J (2019). Weight status at postpartum: being normal weight yet centrally obese!. Food Nutr Sci.

[22] Beyerlein A, Schiessl B, Lack N, Von Kries R (2009). Optimal gestational weight gain ranges for the avoidance of adverse birth weight outcomes: a novel approach. Am J Clin Nutr.

[23] Du MK, Ge LY, Zhou ML, Ying J, Qu F, Dong MY, Chen DQ (2017). Effects of pre-pregnancy body mass index and gestational weight gain on neonatal birth weight. J Zhejiang Univ Sci B.

[24] Nohr EA, Vaeth M, Baker JL, Sørensen TIA, Olsen J, Rasmussen KM (2010). Pregnancy outcomes related to gestational weight gain in women defined by their body mass index, parity, height, and smoking status (American Journal of Clinical Nutrition (2009) 90, (1288–1294)). Am J Clin Nutr.

[25] Sunsaneevitayakul P, Sompagdee N, Asad-Dehghan M, Talungchit BSWP (2022). Effect of Gestational Weight Gain on Overweight and Obese Pregnant Women. Siriraj Med J.

[26] Hill B, McPhie S, Skouteris H (2016). The role of parity in gestational weight gain and postpartum weight retention. Women’s Heal Issues.

[27] Jayasinghe S, Herath MP, Beckett JM, Ahuja KDK, Street SJ, Byrne NM, et al. Gestational weight gain and postpartum weight retention in Tasmanian women: The Baby-bod Study. PLoS One. 2022;17(3 March). 10.1371/journal.pone.0264744. 10.1371/journal.pone.0264744 PMC8939821 35316273

[28] Endres LK, Straub H, McKinney C, Plunkett B, Minkovitz CS, Schetter CD (2015). Postpartum weight retention risk factors and relationship to obesity at 1 year. Obstet Gynecol.

[29] Gondwe A, Ashorn P, Ashorn U, Dewey KG, Maleta K, Nkhoma M (2018). Pre-pregnancy body mass index (BMI) and maternal gestational weight gain are positively associated with birth outcomes in rural Malawi. PLoS One.

[30] Gul R, Iqbal S, Anwar Z, Ahdi SG, Ali SH, Pirzada S. Pre-pregnancy maternal BMI as predictor of neonatal birth weight. PLoS One. 2020;15(10 October):1–9. 10.1371/journal.pone.0240748. 10.1371/journal.pone.0240748 PMC7592734 33112877

[31] Mohapatra I, Harshini N, Samantaray SR, Naik G. Association Between Early Pregnancy Body Mass Index and Gestational Weight Gain in Relation to Neonatal Birth Weight. Cureus. 2022;14(7):0–8. 10.7759/cureus.27089 PMC9391619 36000131

[32] Jeong DE, Hyun SM, Cho I, Lee KN, Ahn K, Ji Kim H (2022). The association between maternal pre-pregnancy body mass index and pregnancy outcomes of preeclampsia. Taiwan J Obstet Gynecol.

[33] Zong X, Wang H, Yang L, Guo Y, Zhao M, Magnussen CG (2022). Maternal pre-pregnancy body mass index categories and infant birth outcomes: a population-based study of 9 million mother-infant pairs. Front Nutr.

[34] Kwon EJ, Kim YJ (2017). What is fetal programming?: A lifetime health is under the control of in utero health. Obstet Gynecol Sci.

[35] Penugonda AJ, Rajan RJ, Lionel AP, Kompithra RZ, Jeyaseelan L, Mathew LG (2022). Impact of exclusive breast feeding until six months of age on common illnesses : a prospective observational study.

[36] Hashemi-Nazari SS, Hasani J, Izadi N, Najafi F, Rahmani J, Naseri P (2020). The effect of pre-pregnancy body mass index on breastfeeding initiation, intention and duration: a systematic review and dose-response meta-analysis. Heliyon..

[37] Ballesta-Castillejos A, Gomez-Salgado J, Gomez-Salgado J, Rodriguez-Almagro J, Ortiz-Esquinas I, Hernandez-Martinez A (2020). Relationship between maternal body mass index with the onset of breastfeeding and its associated problems: an online survey. Int Breastfeed J.

[38] Pinheiro RL, Areia AL, Pinto AM, Donato H (2019). Advanced maternal age: adverse outcomes of pregnancy, a meta-analysis. Acta Med Port.

[39] Lewandowska M, Sajdak S, Więckowska B, Manevska N, Lubiński J (2020). The influence of maternal BMI on adverse pregnancy outcomes in older women. Nutrients.

[40] Sun Y, Shen Z, Zhan Y, Wang Y, Ma S, Zhang S (2020). Effects of pre-pregnancy body mass index and gestational weight gain on maternal and infant complications. BMC Pregnancy Childbirth.

[41] Matijasevich A, Victora CG, Lawlor DA, Golding J, Menezes AMB, Araújo CL (2012). Association of socioeconomic position with maternal pregnancy and infant health outcomes in birth cohort studies from Brazil and the UK. J Epidemiol Commun Health.

[42] Saurel-Cubizolles MJ, Azria E, Blondel B, Regnault N, Deneux-Tharaux C (2022). Exploring the socioeconomic disparities of maternal body mass index: a national study in France. Eur J Public Health.

[43] Arora P, Tamber Aeri B. Gestational weight gain among healthy pregnant women from asia in comparison with Institute of Medicine (IOM) Guidelines-2009: a systematic review. J Pregnancy. 2019;0–10. 10.1155/2019/3849596 PMC6421048 30941218

